# Matrix Metalloproteinase-1 (MMP-1) Promoter Polymorphisms are Well Linked with Lower Stomach Tumor Formation in Eastern Indian Population

**DOI:** 10.1371/journal.pone.0088040

**Published:** 2014-02-05

**Authors:** Sanjib Dey, Nillu Ghosh, Debjit Saha, Kousik Kesh, Arnab Gupta, Snehasikta Swarnakar

**Affiliations:** 1 Drug Development Diagnostic and Biotechnology Division, Indian Institute of Chemical Biology, Kolkata, India; 2 Saroj Gupta Cancer Center and Research Institute, Kolkata, India; Population Health and Preventive Medicine, Malaysia

## Abstract

Expression of matrix metalloproteinase-1 (MMP-1), an interstitial collagenase, plays a major role in cellular invasion during development of gastric cancer, a leading cause of death worldwide. A single-nucleotide polymorphism (SNP) −1607 1G/2G site of the MMP-1 gene promoter has been reported to alter transcription level. While the importance’s of other SNPs in the MMP-1 promoter have not yet been studied in gastric cancer, our aim was to investigate MMP-1 gene promoter polymorphisms and gastric cancer susceptibility in eastern Indian population. A total of 145 gastric cancer patients and 145 healthy controls were genotyped for MMP-1 −1607 1G/2G (rs1799750) by PCR-restriction fragment length polymorphism (RFLP), while MMP-1 −519 A/G (rs1144393), MMP-1 −422 T/A (rs475007), MMP-1 −340 T/C (rs514921) and MMP-1 −320 T/C (rs494379) were genotyped by DNA sequencing. A positive association was found with MMP-1 −422 T/A SNP that showed significant risk for regional lymph node metastasis (*P* = 0.021, Odd’s ratio (OR) = 3.044, Confidence intervals (CI) = 1.187–7.807). In addition, we found a significant association with lower stomach tumor formation among gastric cancer patients for three adjacent polymorphisms near the transcriptional start sites of [MMP-1 −422 T/A (*P* = 0.043, OR = 2.182, CI = 1.03–4.643), MMP-1 −340 T/C (*P* = 0.075, OR = 1.97, CI = 0.94–4.158) and MMP-1 −320 T/C (*P* = 0.034, OR = 2.224, CI = 1.064–40731)]. MMP-1 level in patients’ serum was correlated with MMP-1 promoter haplotypes conferring these three SNPs to evaluate the functional importance of these polymorphisms in lower stomach tumor formation and significant correlation was observed. Furthermore, MMP-1 −519 A/G polymorphism displayed poor cellular differentiation (*P* = 0.024, OR = 3.8, CI = 1.69–8.56) attributing a higher risk of cancer progression. In conclusion, MMP-1 proximal promoter SNPs are associated with the risk of lower stomach tumor formation and node metastasis in eastern Indian population.

## Introduction

Gastric cancer is one of the leading causes of cancer related death in the world [1]. On a global scale, gastric cancer (GC) accounts for approximately 800,000 deaths annually. More than 70% of GC cases occur in developing countries and half the world total occurs in Eastern Asia [1]. It is a leading problem in north-eastern and southern states of the Indian subcontinent [2]. The incidence of gastric cancer varies from country to country, probably as a result of genetic, epigenetic and environmental factors. *Helicobacter pylori* infection is considered as a major risk factor in the development of gastric cancer especially cancer in the lower part (noncardia) of the stomach [3]. A combined analysis of 12 studies of *H. pylori* and gastric cancer estimated that the risk of adenocarcinoma in non-cardia regions of the stomach was nearly six times higher for *H. pylori*-infected people than for uninfected people [3].

Recent studies demonstrate that, carcinogenesis is a multi-cellular and multi-stage process in which destruction of the tissue microenvironment is a requisite for conversion of normal tissue to tumor [4]. Hence, molecular analysis of the tissue microenvironment and its deregulation during neoplasia is a key step to know the mechanisms of malignancy. Matrix metalloproteinases (MMPs), produced by both tumor and normal cells, alter the microenvironment by degrading extracellular matrix, and subsequent cellular signals lead to the early stages of tumor formation [5]. Several of the MMPs have the unique ability to degrade the interstitial collagens (e.g. I, II, and III), the body's most abundant proteins. MMP-1 is the most ubiquitously expressed interstitial collagenase [6] and its overexpression is associated with several pathological conditions, including tumor invasion and metastasis [7]. The overexpression of MMP-1 mRNA has been demonstrated in a variety of cancers such as gastric cancer, colorectal cancer and esophageal cancer [8]–[12]. Overexpression of MMP-1 protein is associated with poor prognosis of esophageal cancer and colorectal cancer [10], [11]. This MMP-1 overexpression may be attributed to the juxtaposition of transcription factor binding sites and cooperativity among the factors that bind to these sites within the promoter region of the MMP-1 gene [13].

The promoter region of MMP-1 contains a guanine insertion/deletion polymorphism (1G/2G polymorphism) at position −1607 which generates the sequence 5′-GGA-3′ which has a 2G allele. The presence of a 2G polymorphism could increase transcriptional activity of endogenous MMP-1 because the guanine insertion creates a binding site for a member of the Ets transcription factor family. The 2G allele may contribute to increased invasiveness of endometrial carcinomas, to the development of ovarian cancer, lung cancer, and colorectal cancer [14]–[21].

Studies in several other genes have provided a new paradigm in which the transcription of a gene is more likely to be influenced by multiple polymorphisms located in the promoter region which act in concert to exert a haplotype effect [22], [23]. Several other single nucleotide polymorphisms (−519A/G, −422T/A, −340C/T, and −320C/T) in the MMP-1 gene promoter have recently been identified [24]. Functional effects of these polymorphisms on the MMP-1 gene promoter activity were assessed in cell lines of melanoma (A2058 and A375), breast cancer (MCF7 and MDA-MB-231), lung cancer (A549 and H69), and colorectal cancer (HT-29, SW-620) by comparing the promoter strengths of the 10 most common haplotypes derived from these polymorphisms [24]. Although the enhanced expression of MMP-1 was associated with local invasion and poor prognosis in gastric cancer [25], the role of the MMP-1 promoter SNPs and their haplotypes in the development of gastric cancer is currently unknown in any human population.

In the present study, we conducted a hospital-based case-control study to explore the association of the MMP-1 gene promoter SNPs [−1607 1G/2G (rs1799750) (MMP-1.1), −519A/G (rs1144393) (MMP-1.2), −422T/A (rs475007) (MMP-1.3), −340C/T (rs514921) (MMP-1.4), and −320C/T (rs494379) (MMP-1.5)] and their haplotypes with the risk of gastric cancer development in an eastern Indian population. We also explored the relationship between the polymorphisms and the clinicopathological factors among gastric cancer patients. The study demonstrated the putative association of MMP-1.3, MMP-1.4, and MMP-1.5 polymorphisms with the risk of lower stomach tumor formation in gastric cancer. The functional importance of MMP-1 polymorphisms in lower stomach tumor formation was confirmed by haplotype effects of these polymorphisms on MMP-1 expression levels in serum. However, no association with the occurrence of gastric cancer was found for any of the above five SNPs of the MMP-1 promoter and their resultant haplotypes.

## Materials and Methods

### Study Subjects: Eastern Indian Case-control Cohort

The eastern Indian case-control cohort consisted of 145 gastric cancer cases and 145 control individuals. The study protocols (2008–2014) were approved by the Ethical Review Boards of the Saroj Gupta Cancer Center and Research Institute, Kolkata, Department of Gastric Surgery, Medical College and Hospital, Kolkata, IPGMER, Kolkata hospitals and the Human Ethics Committee of the Indian Institute of Chemical Biology, Kolkata, India. All patients with a clinical diagnosis of gastric cancer attending the hospital Departments of Gastro-oncology of the Saroj Gupta Cancer Center and Research Institute, Kolkata, the Department of Gastric Surgery, Medical College and Hospital, Kolkata and the IPGMER, Kolkata during June 2008 to May 2013 were identified from hospital registries and contacted during follow up investigations. All participants gave written informed consent for participation. Patients’ demographics, symptoms and tumor grading were recorded and blood samples collected after taking a clinical history and performing clinical and endoscopic examinations. Histological tumor typing was determined on the basis of biopsies or resected specimens. The exclusion criteria included previous history of other metastasized cancer except gastric cancer. Possibility of peptic ulcer without having any cancerous lesion had been excluded by histopathology of stomach lesion. The diagnosis of gastric cancer and TNM staging was based on generally accepted clinical, histological, radiological and immunofluorescence findings and that of the American Joint Committee on cancer (AJCC) and the International Union Against Cancer (UICC) criterion [25]. Individuals who were formerly or who currently have been addicted to tobacco for at least 2 years were defined as tobacco addicted. Persons visiting these hospitals for a routine checkup and who were without a history or diagnosis of any cancer or genetic diseases were also asked to participate and volunteer to donate blood for the study. These subjects were considered healthy controls. All of the cancer patients and control subjects were unrelated and of Indian nationality from West Bengal or the surrounding eastern Indian states. This population was considered representative of an eastern Indian population.

### DNA Extraction and Serum Collection

From each subject, 5 ml of venous blood were drawn aseptically into vacutainer tubes (Qiagen, USA) containing EDTA and stored at 4°C prior to genomic DNA extraction. Genomic DNA was extracted from 3.5 ml of whole-blood within two weeks following sampling using a QIAamp DNA Blood Midi Kit (Qiagen, USA) according to the manufacturer’s protocol. Immediately following blood collection, additional1.5 ml of whole blood was centrifuged at 1800 g for 5 min to obtain the serum fraction. Serum samples were stored at −80°C until analysis.

### Primers and PCR Amplification of the Promoter Region of MMP-1 Genes

Primers were designed with FastPCR software (http://www.biocenter.helsinki.fi) so as to amplify promoter regions of the MMP-1 gene in order to analyze polymorphisms by sequencing (Table 1). PCR was performed in a PCR SPRINT Thermal Cycler (Thermo Electron Corporation, Japan). The target sequence was amplified in a 25-µl reaction volume containing 10–20 ng of genomic DNA, 0.2 mM dNTP, 10 mM Tris-HCl (pH 8.3), 50 mM KCl, 2 mM MgCl2, 0.3 µM of each primer, and 1.0 units of Taq DNA polymerase (Fermentas Taq DNA polymerase, Fermentas, USA). The PCR amplification was carried out with 35 cycles of denaturation at 94°C for 30 s, annealing at 58°C-59°C for 1 min (Table 1) and followed by extension at 72°C for 30 s after the initial activation step of 94°C for 5 min. PCR fragments were analyzed comparing with 100 bp DNA ladder (Fermentas, USA) on an ethidium bromide stained 1.5% agarose gel (Cat No. 014011, Sisco Research Laboratories, India) run for 60 min at 100 V.

**Table 1:** PCR primers used for polymorphism analysis.

Polymorphisms	Primer sequence (5′-3′)	Ta (°C)	Product size	Screening method used
MMP-1 −1607 1G/2G	ACATTGCAGGATGTGCAGGCTCTT (F)	58	782 bp	Sequencing
	CTTGGGTACTGGTGACCGGTGTCA (R)			
MMP-1 −1607 1G/2G	TGACTTTTAAAACATAGTCTATGTTCA (F)	58.5	269 bp	PCR-RFLP
	TCTTGGATTGATTTGAGATAAGTCATAGC (R)			
MMP 1 −519 A/G	TACAGGTGCATGACTCCATGCTTG (F)	58	885 bp	Sequencing
	TCTAGAGTCGCTGGGAAGCTGTGA (R)			
MMP-1 −422 T/A	TACAGGTGCATGACTCCATGCTTG (F)	58	885 bp	Sequencing
	TCTAGAGTCGCTGGGAAGCTGTGA (R)			
MMP-1 −340 T/C	TACAGGTGCATGACTCCATGCTTG (F)	58	885 bp	Sequencing
	TCTAGAGTCGCTGGGAAGCTGTGA (R)			
MMP-1 −320 T/C	TACAGGTGCATGACTCCATGCTTG (F)	58	885 bp	Sequencing
	TCTAGAGTCGCTGGGAAGCTGTGA (R)			

### Genotyping

For genotyping by sequencing, any unincorporated dNTPs were dephosphorylated and unincorporated primers were removed from the PCR product by shrimp alkaline phosphatase (Fermentas, USA) and exonuclease-I enzyme (Fermentas, USA). PCR products were used for sequencing with BigDye® Terminator v3.1 Cycle Sequencing Kit (Applied Biosystems, USA) and sequenced using a ABI PRISM 3100 genetic analyzer (Perkin-Elmer ABI, Foster City, Calif.) according to the manufacturer’s protocol. The PCR amplicon was sequenced in both directions with forward and reverse PCR primers eliminating the possibility of compression artifacts. Sequencing chromatograms were analyzed using Sequence Scanner Software v1.0 (Applied Biosystems, USA) to analyze alterations of the nucleotides and thus genotypes.

The MMP-1.1 1G/2G polymorphism was genotyped by PCR*-* restriction fragment length polymorphism (RFLP) method using *AluI* enzyme (New England BioLabs, Inc. (NEB); Ipswich, MA), as described previously [26].

### Enzyme-Linked Immunosorbent Assay (ELISA)

Serum MMP-1 protein level (pro and active) in patients with upper stomach cancer with non risk haplotypes, and in lower stomach cancer with risk haplotypes (contains one or more risk alleles) were compared using a commercially available MMP-1 ELISA kit (ab10063, Abcam, USA), according to the manufacturer’s instruction. Serum samples were diluted (1∶5 v/v) in assay diluent provided in the kit. Briefly, 100 µl of the standards, blanks and diluted serum sample were pipetted into the anti-human MMP-1 precoated 96-well plate provided within the kit and incubated for 2.5 hours at 37°C. After several washes, biotinylated anti-human MMP-1 antibody (diluted 1∶1000) was added to the wells and the plate was incubated for 1 hour at room temperature. After washing away unbound biotinylated antibody, HRP-conjugated streptavidin is pipetted to the wells. The plate was again incubated for 45 min. After extensive washing, 100 µl of the substrate tetramethylbenizidine was pipette into each well, and the plate was incubated for 30 min in the dark at room temperature (25°C). A stop solution was added upon completion. Each sample was tested in duplicate. The absorbance values of the blanks, samples, and standards were read on a microplate reader at a wavelength of 450 nm. The level of MMP-1 protein in the samples was obtained by comparison with the standard curve, generated from standard supplied by the manufacture. The minimum detectable level of MMP 1 was 8 pg/ml.

### Statistical Analysis

Statistical analysis was performed using the SPSS software (version 16.0J. SPSS, Inc., Chicago, IL, USA). Significant differences between age at interview for controls and age at diagnosis for cancer cases were assessed using the Student’s t-test for comparison of means using GraphPad InStat3 software (GraphPad Software, Inc., San Diego California USA). Hardy–Weinberg equilibrium (HWE) analyses were performed to compare observed and expected allele frequencies using a chi-square test for controls to ensure that each marker was in equilibrium (*p*>0.05). The minor allele frequency (MAF) (*–model* option) and HWE (*–hardy* option) for each SNP was estimated from the control population using Plink v0.99 [27]. Case-control data were analyzed using two-sided 2-by-2 or 2-by-3 contingency tables according to the genotype by the Pearson chi-square test. The odds ratio (OR) and 95% confidence interval (CI) of the genotypes were calculated from a multivariate logistic regression model adjusted for age (continuous variable), sex and tobacco addiction. In this study, we defined that the 1G allele of the 1G-1607 2G SNP, the A allele of the A-519G SNP, the A allele of the T-422A SNP, the T allele of the T-340C SNP and the T allele of the T-320C SNP were reference alleles. The results were evaluated with above alleles as a reference using the multinomial logistic regression model. In analyzing the relationship between the SNP genotypes and disease status of GC, the stage of cancer, histological classification and depth of tumor invasion were transformed to binary data (Stage I+II vs. stage III+IV, well-differentiated+moderately-differentiated vs. Poorly-differentiated, and T1+T2 vs. T3+T4). The relationship between genotype distributions and tumor depth or stage was also examined using a multinomial logistic regression model adjusted for age (continuous variable), sex and tobacco addiction as a potential confounding factor. Haplotype frequencies were analyzed using the program Haploview (ver. 4.1, Broad Institute, Chembridge, USA) [28]. All results were considered statistically significant if the *p* value was <0.05. A post hoc power calculation has been performed to test the statistical power of the present study according to the method of Schlesselman, JJ [29].

## Results

### Description of the Study Population

The study population consisted of 145 gastric cancer patients having 112 (77.2%) males and 33 (22.8%) females with an age range of 42.6–65.8 years, as well as 145 control subjects having 81 (55.9%) males and 64 (44.1%) females with an age range of 34.5–62.5 years. Patients and control subjects were derived from the same geographic location and are representative of an eastern Indian population. There were statistically significant differences in the distribution of gender (*p*<0.001) and age (*p*<0.0001) between patients and controls. Also, significantly more tobacco-addicted individuals (p<0.0001) were present among patients (68.3%) compared with controls (28.3%). So, in the course of risk estimation, an age, sex and addiction-adjusted Odds ratios were calculated.

### Detection of Single Nucleotide Polymorphisms in the MMP-1 Promoter

Naturally occurring, common sequence variants of the MMP-1 gene promoter in cases and controls were searched by direct DNA sequencing. Alignment of sequence chromatogram with the MMP-1 gene promoter contig sequence (Genebank accession no- AF023338) confirmed the SNP positions at −1607 (1G/2G, i.e., G insertion/deletion), −519 (A/G), −422 (T/A), −340 (T/C) and −320 (T/C) (Figure S1 in File S1). MMP-1.1 genotyping by PCR-RFLP was then performed among the gastric cancer patients and controls. Figure S1A (File S1) shows a typical PCR-RFLP pattern. The 269 bp target region of the MMP-1 gene promoter was PCR-amplified and digested with *AluI*, which cleaved the 1G allele to generate two fragments of 241 bp and 28 bp. The 2G allele did not digest with *AluI*. Heterozygous genotype showed three bands of 269 bp, 241 bp and 28 bp (Figure S1A in File S1). MMP-1.2, MMP-1.3, MMP-1.4) and MMP-1.5 were genotyped by DNA sequencing (Figure S1B in File S1). The MMP-1.1 genotype analysis by DNA sequencing was restricted to a pilot study as well as for 10% of the population for rechecking. All genotyping was performed among 145 patients and 145 controls.

### Association between Individual SNPs of MMP-1 Promoter and Gastric Cancer Risk

The genotype distributions of MMP-1 polymorphisms were consistent with the Hardy–Weinberg equilibrium (HWE). The study would have reached a power of 80% (Z-alpha = 1.645, for alpha = 0.05) with 145 cases, case control ratio of 1.0 and 10% exposed control to the risk allele having OR of 2.31.

MMP-1.1 polymorphism showed no significant difference in distribution of genotypes (1G1G vs. 1G2G, 2G2G and 1G2G+2G2G) between patients and controls (*p* = 0.430, *p* = 0.465 and *p* = 0.411 respectively). Also, the allele frequency distribution (1G vs. 2G) was not significant between patients and controls (*p* = 1.000), and thus did not confer any risk for gastric cancer development (Table 2). It is also noteworthy that the 2G allele was the major allele in this population and we took it as a risk allele as many other studies have previously considered it a risk allele [14], [30], [31].

**Table 2:** Analysis of association between MMP-1 SNPs and the risk of occurrence of gastric cancer.

Genotype	GC Patient		Controls		OR	95% CI	P value
	n	%	N	%			
**MMP-1.1 (−1607 1G/2G)**	**145**		**145**				
**1G1G**	23	15.9	20	13.8	1 (Ref)		
**1G2G**	66	45.5	72	49.7	0.742	0.353–1.559	0.430
**2G2G**	56	38.6	53	36.6	0.746	0.340–1.637	0.465
**1G2G+2G2G**	122	84.1	125	86.3	0.742	0.364–1.511	0.411
**1G allele**	112	38.6	112	38.6	1 (Ref)		
**2G allele**	178	61.4	178	61.4	1.000	0.716–1.397	1.000
**MMP-1.2 (−519 A/G)**	**145**		**145**				
**AA**	91	62.8	100	69.0	1 (Ref)		
**AG**	49	33.8	41	28.3	1.154	0.672–1.982	0.603
**GG**	5	3.4	4	2.8	1.640	0.382–7.047	0.506
**AG+GG**	54	37.2	45	31.0	1.194	0.705–2.020	0.510
**A allele**	231	79.7	241	83.1	1 (Ref)		
**G allele**	59	20.3	49	16.9	1.256	0.826–1.911	0.337
**MMP-1.3 (−422 T/A)**	**145**		**145**				
**TT**	17	11.7	17	11.7	1.221	0.547–2.725	0.626
**TA**	53	36.6	51	35.2	1.000	0.581–1.722	0.999
**AA**	75	51.7	77	53.1	1 (Ref)		
**TA+TT**	70	48.3	68	46.9	1.065	0.646–1.755	0.806
**T allele**	87	30	85	29.3	1.034	0.724–1.476	0.927
**A allele**	203	70	205	70.7	1 (Ref)		
**MMP-1.4 (−340 T/C)**	**145**		**145**				
**TT**	69	47.6	78	53.8	1 (Ref)		
**TC**	64	44.1	57	39.3	1.209	0.719–2.032	0.474
**CC**	12	8.3	10	6.9	1.499	0.556–4.043	0.424
**TC+CC**	76	52.4	67	46.2	1.246	0.757–2.051	0.388
**T allele**	202	69.7	213	73.4	1 (Ref)		
**C allele**	88	30.3	77	26.6	1.205	0.839–1.73	0.357
**MMP-1.5 (−320 T/C)**	**145**		**145**				
**TT**	73	50.3	67	46.2	1 (Ref)		
**TC**	61	42.1	64	44.1	0.769	0.454–1.303	0.329
**CC**	11	7.6	14	9.7	0.650	0.259–1.631	0.358
**TC+CC**	72	49.7	74	53.8	0.734	0.443–1.214	0.228
**T allele**	207	71.4	198	69.3	1 (Ref)		
**C allele**	83	28.6	92	31.7	0.863	0.605–1.231	0.469

Adjusted OR calculated for age, sex and addiction, by binary logistic regression model using SPSS v16.0 software. P value is for χ^2^ test showing the significance of difference in the distributions of the genotypes and alleles between patients and controls. OR = Odds ratio, CI = Confidence Interval, Ref = Reference genotype or allele to calculate OR.

In gastric cancer patients, the frequency of the MMP-1.2 AG, GG and AG+GG genotypes were not significantly different from healthy controls (*p* = 0.603, *p* = 0.506 and *p* = 0.510 respectively) and thus did not confer any significant risk for gastric cancer. Also, the allele frequency (A vs. G) in cancer patients were not significantly differed from healthy controls (*p* = 0.337) and did not confer risk for gastric cancer development (Table 2).

The genotype frequency distribution, (AA vs. TT, TA and TA+TT) of the MMP-1.3 polymorphism in the patient population was not significantly different from healthy controls (*p* = 0.626, *p* = 0.999 and *p* = 0.806 respectively) and thus did not confer any significant risk for gastric cancer. Also, the allele frequency (T vs. A) distribution in cancer patients and controls were not significant (*p* = 0.927) (Table 2).

For patient MMP-1.4 polymorphism, genotypes (TT vs. TC, CC and TC+CC) showed a similar distribution to that seen in controls (*p* = 0.474, *p* = 0.424 and *p* = 0.388 respectively) showing no association for gastric cancer risk. Also, the distribution of allele frequency (T vs. C) in patients and controls was not statistically different (*p* = 0.357), further showing the absence of any association with gastric cancer risk (Table 2).

MMP-1.5 polymorphism genotype frequencies (TT vs. TC, CC and TC+CC) in patients was similarly distributed to that of controls (*p* = 0.329, *p* = 0.358 and *p* = 0.228 respectively) showing no significant risk for gastric cancer. Also, the distribution of allele frequency (T vs. C) in both patients and controls was not significantly different (*p* = 0.469) (Table 2).

### Association between Individual SNPs of MMP-1 as well as Demographic and Clinicopathological Features at the Time of Gastric Cancer Diagnosis

Associations were examined between each polymorphism and the demographic and clinicopathological features of gastric cancer patients and controls (Table 3). MMP-1.1, MMP-1.2, MMP-1.3, MMP-1.4 and MMP-1.5 polymorphisms did not play any role in determining gastric cancer risk for any age group, gender or tobacco-addiction status.

**Table 3:** Association between the genotypes of MMP-1 SNPs and clinicopathological characteristics of gastric cancer patients.

	MMP-1.1(−1607 1G/2G), n (%)		MMP-1.2(−519 A/G), n (%)		MMP-1.3(−422 T/A), n (%)		MMP-1.4(−340 T/C), n (%)		MMP-1.5(−320 T/C), n (%)	
	1G/1G	1G/2G+2G/2G	A/A	A/G+G/G	A/A	T/A+T/T	T/T	T/C+CC	T/T	T/C+CC
**Age (years)**										
*≥45* (Case/Cont)	18/8 (15.0/11.0)	102/65 (85.0/89.0)	72/49 (60.0/67.1)	48/24 (40.0/32.9)	61/38 (50.8/52.1)	59/35 (49.2/47.9)	58/38 (48.3/52.1)	62/35 (51.7/47.9)	56/32 (46.7/43.8)	64/41 (53.3/56.2)
OR (95% CI) (a)	1 (Ref)	0.77 (0.31–1.90)	1 (Ref)	1.38 (0.75–2.55)	1 (Ref)	1.116 (0.62–2.02)	1 (Ref)	1.13(0.63–2.04)	1 (Ref)	0.90 (0.50–1.62)
*<45* (Case/Cont)	5/12 (20.0/16.7)	20/60 (80.0/83.3)	19/51 (76.0/70.8)	6/21 (24.0/29.2)	14/39 (56.0/54.2)	11/33 (44.0/45.8)	11/40 (44.0/55.6)	14/32 (56.0/44.4)	17/35 (68.0/48.6)	8/37 (32.0/51.4)
OR (95% CI) (a)	1 (Ref)	0.56 (0.16–1.97)	1 (Ref)	0.78 (0.26–2.30)	1 (Ref)	0.90 (0.35–2.33)	1 (Ref)	1.62 (0.63–4.19)	1 (Ref)	0.42 (0.16–1.14)
**Gender**										
*Male* (Case/Cont)	17/12 (15.2/14.8)	95/69 (84.8/85.2)	69/58 (61.6/71.6)	43/23 (38.4/28.4)	58/47 (51.8/58.0)	54/34 (48.2/42.0)	51/44 (45.5/54.3)	61/37 (54.5/45.7)	57/36 (50.9/44.4)	55/45 (49.1/55.6)
OR (95% CI) (b)	1 (Ref)	1.07 (0.47–2.44)	1 (Ref)	1.45 (0.77–2.74)	1 (Ref)	1.32 (0.73–2.41)	1 (Ref)	1.41 (0.78–2.55)	1 (Ref)	0.69 (0.38–1.27)
*Female* (Case/Cont)	6/8 (18.2/12.5)	27/56 (81.8/87.5)	22/42 (66.7/65.6)	11/22 (33.3/34.4)	17/30 (51.5/46.9)	16/34 (48.5/53.1)	18/34 (54.5/53.1)	15/30 (45.5/46.9)	16/31 (48.5/48.4)	17/33 (51.5/51.6)
OR (95% CI) (b)	**1 (Ref)**	**0.18 (0.04–0.85)**	1 (Ref)	0.76 (0.29–2.02)	1 (Ref)	0.60 (0.23–1.55)	1 (Ref)	0.94 (0.38–2.37)	1 (Ref)	0.83 (0.33–2.09)
**Location of cancer**										
Lower stomach/Upper stomach	16/7 (18.0/15.6)	73/38 (82.0/84.4)	52/31 (58.4/68.9)	37/14 (41.5/31.1)	42/29(47.2/64.4)	47/16 (52.8/35.5)	37/26 (41.6/57.8)	52/19 (58.4/42.2)	37/28 (41.6/62.2)	52/17 (58.4/37.8)
OR (95% CI) (c)	1 (Ref)	0.87 (0.33–2.31)	1 (Ref)	1.73 (0.79–3.77)	1 (Ref)	**2.18 (1.03–4.64)**	1 (Ref)	**1.97 (0.94–4.15)**	1 (Ref)	**2.22 (1.06–4.07)**
**Tumor size**										
≥5 cm/<5 cm	7/16 (11.3/19.3)	55/67 (88.7/80.7)	44/47 (71.0/56.6)	18/36 (29.0/43.4)	30/45 (48.4/54.3)	32/38 (51.6/45.7)	29/40 (46.8/48.2)	33/43 (53.2/51.8)	31/42 (50.0/50.6)	31/41 (50.0/49.4)
OR (95% CI) (c)	1 (Ref)	1.86 (0.71–4.88)	1 (Ref)	0.54 (0.26–1.1)	1 (Ref)	1.31 (0.67–2.57)	1 (Ref)	1.01 (0.51–1.99)	1 (Ref)	1.11 (0.57–2.18)
Histological classification (*)										
PD/WD+MD	11/11 (13.9/19.3)	68/46 (86.1/80.7)	42/44 (53.2/77.2)	37/13 (46.8/22.8)	43/29 (54.4/50.9)	36/28 (45.6/49.1)	39/25 (49.4/43.9)	40/32 (50.6/56.1)	39/30 (49.4/52.6)	40/27 (50.6/47.4)
OR (95% CI) (c)	1 (Ref)	1.60 (0.63–4.10)	1 (Ref)	**3.8 (1.69–8.56)**	1 (Ref)	1.31 (0.67–2.57)	1 (Ref)	0.85 (0.42–1.74)	1 (Ref)	1.21 (0.59–2.46)
**Depth of invasion (#)**										
T3+T4/T1+T2	10/13 (18.5/14.3)	44/78 (81.5/85.7)	35/56 (64.8/61.5)	19/35 (35.2/38.5)	26/49 (48.1/53.8)	28/42 (51.9/56.2)	29/40 (53.7/44.0)	25/51 (46.3/56.0)	26/47 (48.1/51.6)	28/44 (51.9/48.4)
OR (95% CI) (c)	1 (Ref)	0.75(0.30–1.86)	1 (Ref)	0.99 (0.48–2.05)	1 (Ref)	1.37 (0.68–2.76)	1 (Ref)	0.73 (0.36–1.46)	1 (Ref)	1.18 (0.5842.37)
**Regional Lymph Node Metastasis**										
N+ve/N-ve	18/5 (15.1/19.2)	101/21 (84.9/80.8)	76/15 (63.9/57.7)	43/11 (36.1/42.3)	56/19 (47.1/73.1)	63/7 (52.9/26.9)	62/7 (52.1/26.9)	57/19 (47.9/73.1)	59/14 (49.6/53.8)	60/12 (50.4/46.2)
OR (95% CI) (c)	1 (Ref)	1.32 (0.44–3.98)	1 (Ref)	0.75 (0.31–1.81)	1 (Ref)	**3.04 (1.19–7.81)**	1 (Ref)	**0.34 (0.13–0.88)**	1 (Ref)	1.14 (0.48–2.68)
**Distant Metastasis**										
Yes/No	6/17 (12.8/17.3)	41/81 (87.2/82.7)	31/60 (66.0/61.2)	16/38 (34.0/38.8)	27/48 (57.4/49.0)	20/50 (42.6/51.0)	28/41 (59.6/41.8)	19/57 (40.4/58.2)	23/50 (48.9/51.0)	24/48 (51.1/49.0)
OR (95% CI) (c)	1 (Ref)	1.54 (0.55–4.33)	1 (Ref)	0.87 (0.41–1.85)	1 (Ref)	0.71 (0.35–1.46)	1 (Ref)	**0.49 (0.24**–**1.03)**	1 (Ref)	1.12 (0.55–2.29)
**TNM classification**(¥)										
Stage III+IV/I+II	13/10 (14.8/17.5)	75/47 (85.2/82.5)	59/32 (67.0/56.1)	29/25 (33.0/43.9)	43/32 (48.8/56.1)	45/25 (51.2/43.9)	46/23 (52.3/40.4)	42/34 (47.7/59.6)	42/31 (47.7/54.4)	46/26 (52.3/45.6)
OR (95% CI) (c)	1 (Ref)	1.30 (0.52–3.25)	1 (Ref)	0.66 (0.33–1.32)	1 (Ref)	1.36 (0.69–2.68)	1 (Ref)	0.63 (0.32–1.25)	1 (Ref)	1.37 (0.69–2.72)

P value, ORs and 95% CIs were calculated by unconditional binary logistic regression (using SPSS v16 software) with respective reference genotypes. (a) Adjusted with Sex. (b) Adjusted with Age. (c) Adjusted with Age, Sex, and Addiction. Values in bold indicate positive significance (P<0.05). (*) Histological classification is dependent on the basis of glandular architecture of adenocarcinoma. WD = Well differentiated, MD = Moderately differentiated, PD = poorly differentiated. (¥) Tumor stage is classified according to the criterion of the American Joint Committee on Cancer (AJCC) and the International Union Against Cancer (UICC) TNM stage grouping [25]. (#) Depth of tumor defined according to the criterion of AJCC.

MMP-1.1 and MMP-1.2 polymorphisms did not confer any significant risk for tumor location or degree of tumor progression in gastric cancer (Table 3). However patients carrying combination of AG and GG genotype of MMP-1.2 polymorphism were significantly distributed among histological subtypes of cancer and showed significantly greater risk for poorly differentiated (PD) carcinomas (*p* = 0.001, OR = 3.803, CI = 1.69–8.56) (Table 3).

For MMP-1.3 polymorphism, patients carrying combination of TA and TT genotypes were at more risk of lower stomach cancer (lower & middle body, antrum and pylorus of stomach) (*p* = 0.043, OR = 2.18, CI = 1.03–4.64). Also, the combination of TA and TT genotypes were found more frequently in gastric cancer patients with 10 or more metastatic lymph nodes (*p* = 0.021, OR = 3.044, CI = 1.187–7.807), suggesting that the T allele had a detrimental effects on gastric cancer progression and early metastasis (Table 3).

For MMP-1.4 polymorphism, the distribution of TC and CC genotypes among patients with different tumor locations in the stomach was close to reaching a statistical significant association with the risk of lower stomach cancer (*p* = 0.075, OR = 1.969, CI = 0.94–4.15) (Table 3). Contradictory to MMP-1.3 polymorphism, patients carrying a combination of TC and CC genotypes gave protection against regional lymph node metastasis (*p* = 0.026, OR = 0.34, CI = 0.13–0.88) in addition to distant metastasis (*p* = 0.061, OR = 0.49, CI = 0.24–1.03) of gastric cancer (Table 3).

For MMP-1.5 polymorphism, a combination of TC and CC genotypes showed a significant association with location of stomach tumor. Patients carrying a combination of TC and CC genotypes were at more risk of lower stomach cancer (*p* = 0.034, OR = 2.22, CI = 1.06–4.07) (Table 3).

In stratification analysis for SNPs and lower stomach tumor formation, the study would have reached a power of 80% (Z-alpha = 1.645, for alpha = 0.05) with 89 lower stomach cases, lower stomach and upper stomach sample ratio of 0.505 and 35% exposed upper stomach patients to the risk allele having OR of 2.5.

### Genotype Frequency at Two Linked Loci of MMP-1 SNPs and Risk to Gastric Cancer

To evaluate the combined effects of two linked loci on the risk of cancer, as individual SNPs did not confer risk for gastric cancer development, the combined genotype frequencies were compared in patients and controls. Distributions of paired loci frequencies for adjacent polymorphisms were observed with increasing order of variant allele and associations were examined. There was no dose dependent association observed, the number of variant allele was increased for any combination of adjacent paired loci (Table 4).

**Table 4:** Distribution of MMP-1 paired loci polymorphisms with increasing order of variant alleles and association with the gastric cancer risk.

MMP-1.1-1.2	Con	Patient	OR	95% CI	P
0	13 (9.0)	16 (11.0)	1 (Ref)		
1	49 (33.8)	37 (25.5)	0.529	0.201–1.390	0.286
2	71 (49.0)	76 (52.5)	0.576	0.231–1.431	0.329
3	12 (8.3)	16 (11.0)	0.727	0.231–2.285	0.798
4	0	0	*		
**MMP-1.2-1.3**					
0	49 (33.8)	45 (31.0)	1 (Ref)		
1	67 (46.2)	64 (44.2)	0.948	0.537–1.674	0.885
2	20 (13.8)	26 (17.9)	1.04	0.501–2.157	1
3	9 (6.2)	10 (6.9)	0.8	0.300–2.131	0.803
4	0	0	*		
**MMP-1.3-1.4**					
0	45 (31.0)	39 (26.9)	1 (Ref)		
1	54 (37.2)	57 (39.3)	0.984	0.535–1.815	1
2	30 (20.7)	31 (21.4)	0.985	0.486–1.999	1
3	16 (11.0)	16 (11.0)	0.848	0.363–1.98	0.829
4	0	2 (1.4)	0.53	0.083–3.373	0.653
**MMP-1.4-1.5**					
0	27 (18.6)	25 (17.2)	1 (Ref)		
1	67 (46.2)	69 (47.6)	0.936	0.468–1.873	1
2	51 (35.2)	51 (35.2)	0.861	0.419–1.768	0.718
3	0	0	*		
4	0	0	*		

OR, odds ratio; CI, confidence interval; Ref, reference.

0 = no risk allele for both loci, 1 = one risk allele for any one loci, 2 = two risk allele for both the loci, 3 = one risk allele for any one locus and two risk allele for another locus, 4 = All risk allele for both the loci.

Adjusted OR calculated for age, sex, addiction, by binary logistic regression model using SPSS v16.0 software.

P value is for χ^2^-test showing the significance of difference in the distributions of the variant alleles between patients and controls.

Values in bold indicate positive significance (P<0.05).

No risk allele for both the gene was taken as reference to calculate OR.

(*) OR not calculated because of very low frequency in both control and patient population.

### Linkage Disequilibrium between the SNPs of MMP-1 Promoter and Haplotype Frequencies with the Susceptibility to Gastric Cancer

**Figure 1:**
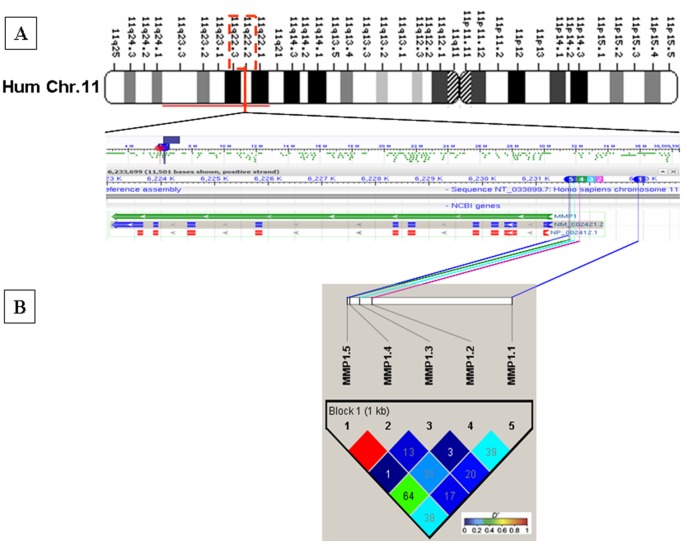
Gene map and LD structure of MMP-1 promoter locus. (A) SNP positions in the gene map of MMP-1 gene (prepared using NCBI sequence viewer) in human chromosome 11. Different color schemes are assigned to show each SNP position in the map by marking the map according to the reference assembly (HuRef NCBI Build 36.7) position of the SNP in the chromosome. The number in different colored boxes indicates the SNP alias number used in our study. (B) LD structure of MMP-1 promoter locus SNPs. Linkage disequilibrium plots follows the GOLD heat map Haploview 4.2 color scheme. Block definition is based on the method of Gabriel et al. [32]. Haploview plot of MMP-1 SNPs genotyped in 290 subjects (145 patients and 145 controls common for all SNPs studied) in our study. Numbers in squares are pair wise D' values between SNPs.

All the polymorphisms in the MMP-1 gene promoter were assessed for linkage disequilibrium (LD) between the polymorphisms and to identify common haplotypes present in the patient and control cohorts. The region showed low to substantial LD between the polymorphisms, except MMP-1.4 and MMP-1.5 which were in complete LD in the case-control study cohort (Figure 1) (The linkage disequilibrium block definitions were based on the method of Gabriel *et al.*) [32]. Sixteen haplotypes were identified with a frequency higher than 1% both in control and patient population, including all the five markers covering the MMP-1 gene promoter. The extended haplotype frequency distributions of MMP-1 polymorphisms were not significant between gastric cancer patients and controls and none of these haplotypes conferred risk for gastric cancer occurrence (data not shown).

### Functional Haplotype Effects of MMP-1 Polymorphisms’ on Lower Stomach Tumor Formation

Sixteen haplotypes were identified with a frequency higher than 1% and categorized by increasing order of polymorphic variant alleles and analyzed for gene-dosage effect (Table S1 in File S1). The difference in the haplotype frequency was significant between lower stomach gastric cancer patients and upper stomach gastric cancer patients as the number of polymorphic variant alleles were increased resulting in the increase of risk for lower stomach gastric cancer development with highest OR of 2.155, 95% CI = 1.317–3.526, P = 0.0028, χ^2^ = 9.52 for combined haplotypes containing 4 risk alleles (Table 5).

**Table 5:** Association of increasing order of MMP-1 polymorphic variant alleles in haplotypes with Lower stomach gastric cancer risk.

Haplotype	Upper Stomach	Lower Stomach	OR	95% CI	P	χ^2^ (Pearson)
0	0.138	0.091	Ref			
1	0.336	0.379	1.711	1.264–2.315	0.0005	12.22
2	0.328	0.298	1.378	1.013–1.874	0.0439	4.18
3	0.159	0.177	1.688	1.201–2.373	0.0027	9.15
4	0.038	0.054	2.155	1.317–3.526	0.0028	9.52
5	0	0				

Haplotypes order as MMP-1.1-MMP-1.2-MMP-1.3-MMP-1.4-MMP-1.5.

(a) two sided χ^2^ Association P value, OR = Odds ratio, CI = Confidence Interval, Ref = Reference haplotype to calculate OR. Other possible combinations of linked loci are omitted because of null frequency in the population. Haplotype frequency and haplotype association test performed using Haploview4.2 software. Haplotype numbers denote the number of risk allele(s) present in the haplotypes.

**Figure 2:**
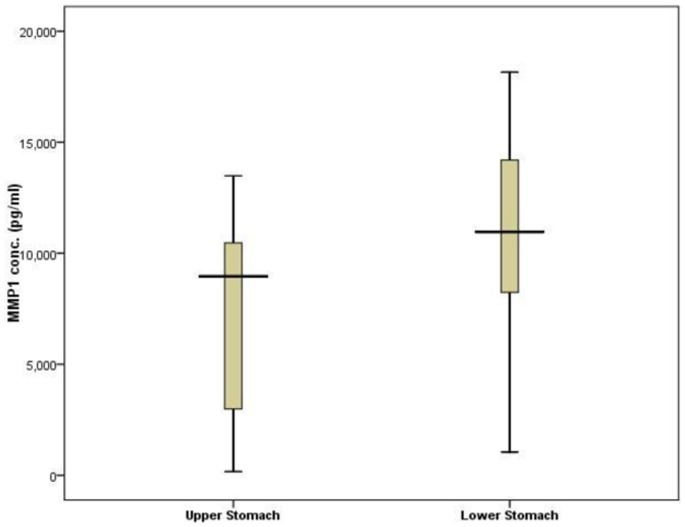
Haplotype effect on serum MMP-1 concentration. Serum MMP-1 level in patients with lower stomach cancer with risk haplotypes (n = 54), and upper stomach cancer with non risk haplotypes (n = 15) were compared by ELISA. Results showed patients with lower stomach cancer exhibit a 1.43 fold higher MMP-1 level than in upper stomach cancer patients (p<0.05 by t-test) (box whisker diagram).

In order to investigate the impact of MMP-1 promoter polymorphisms on the gene function, we compared serum MMP-1 level by serum ELISA in patients having lower stomach cancer with combined risk haplotypes (having at least any one risk allele) vs. upper stomach cancer with the reference haplotype (1G-A-A-T-T). The serum MMP-1 protein concentration was almost 1.5 fold higher in patients with lower stomach cancer (n = 54) (with combined risk haplotypes) than patients with upper stomach cancer (n = 15) (with reference haplotype; p = 0.024), suggesting the functional importance of these SNPs in the regulation of MMP-1 gene expression and there by influencing the lower stomach carcinogenesis process (Figure 2). The analysis has been repeated and confirmed upon log transforming the data (Figure S2 in File S1).

## Discussion

Although MMPs are not oncogenic or mutagenic, they alter the microenvironment and may affect the process of carcinogenesis and its histology, and appear to be induced at the level of transcriptional activation [33]. Being a member of MMP family, MMP-1 has been reported to play an important role in cancer invasion through overexpression, which is associated with metastasis and poor prognosis in esophageal cancer, ovarian cancer, cutaneous malignant melanoma, colorectal cancer and gastric cancer [11], [15], [16], [25], [34]. Diffuse types of gastric cancer are usually characterized by an abundant deposition of collagen fibers, possibly requiring higher levels of MMP-1 expression for proper tissue remodeling of the microenvironment [30]. The genetic background has been suggested to play an important role in the incidence and progression of gastric cancer [30]. In addition, some polymorphic genes encoding metabolic enzymes and cell cycle regulators, such as methylene tetrahydrofolate reductase, NADPH: quinone oxidoreductase and Cyclin D1 have been documented to confer a susceptibility to gastric cancer [35]–[37]. Therefore, polymorphic genes, alone, in combination with others or through interaction with exogenous risk factors, may be used as predicative parameters for screening individuals at a high risk of gastric cancer.

To the best of our knowledge, this study of the association of MMP-1 variants and the risk of gastric cancer development and progression in an eastern Indian population is the first of its kind with a focus on a −1607 1G/2G polymorphism (MMP-1.1), and an additional four polymorphisms between this SNP and the transcription start site. MMP-1 promoter region functional polymorphisms responsible for its expressional alterations have been correlated with various disease processes. Despite this, in our study MMP-1.1 polymorphism and additionally four other SNPs (MMP-1.1, MMP-1.2, MMP-1.3 and MMP-1.4) in the promoter region are not correlated with gastric cancer occurrence, suggesting these promoter variants to be low penetrance risk factors in gastric cancer. The mitogen-activated protein kinase signaling pathway regulates MMP-1 gene expression by activating cofactors that interact with AP1 and polyoma-enhancing activity–3/E26 virus (PEA3/Ets) transcription factor-binding sites located within the promoter region [38]. It is well known that the 2G type of SNP at −1607 (MMP-1.1) in the promoter of MMP-1 creates a core recognition sequence (5′-GGAT-3′) that represents the binding site for Ets family transcription factors. The promoter containing the 2G allele displays significantly higher transcriptional activity than the 1G allele in normal fibroblasts and melanoma cells [13]. Also, Ets transcription factors can positively and negatively activate transcription by interaction with coregulatory-binding partners or by regulating phosphorylation [38]. This suggests that the Ets family proteins and partner proteins may differ in various cell types [38]. Alternatively, other pathways regulating MMP-1 expression may act independently of the SNP at −1607 bp [39]. Similar results for MMP-1.1 polymorphism have been reported in other investigations on gastric cancer using Japanese populations, gastric cardiac adenocarcinoma (GCA) in a Chinese population, and prostate cancer in a Turkish population. However contradictory results have been reported in other investigations on colorectal cancer using Japanese, Korean and Italian populations [15]–[17], [30], [31], [40]. The 2G allele appears to be more frequently identified in Asian populations than in European populations [16], [30], [41], and this is also supported by our data. These discordant results may be a consequence of the number of subjects, the ethnicity of the population and the source of DNA (tumor derived compared to normal genomic) [20].

Patients having combination of the TA and TT genotype for MMP-1.3 polymorphism, a combination of the TC and CC genotype for MMP-1.4 polymorphism, and a combination of the TC and CC genotype for MMP-1.5 polymorphism are at more risk of lower stomach cancer (lower & middle body, antrum and pylorus of stomach), suggesting that these three polymorphisms, separately or in combination may be used as a marker for diagnosing and treating patients having lower stomach cancer in our population. In India, a trend towards an increase in the incidence of cardia tumors (upper stomach cancer) was observed from different cancer registries. Contradictory results were found for our population showing higher incidence of noncardia cancers (lower stomach cancer) (64%) suggesting Indian cancer registries may have inadequate data regarding eastern Indian populations, especially for the region of West Bengal, Bihar and Orissa. Further, these observed differences between gastric cancers by anatomic site and risk of lower stomach cancer incidence with respect to three adjacent SNPs near the transcription start site of the MMP-1 gene suggest that they are distinct diseases with different epidemiological etiologies which may be governed by these polymorphisms in the eastern Indian region. Detailed epidemiological analyses of their demographic trends and risk factors in other cancer types in similar populations will help us to apply new strategies to control cancer. Also, no previous information is available for these polymorphisms in the occurrence of gastric cancer. So, further studies need to confirm our findings in larger sample sizes and in other populations.

In chondrosarcoma, esophageal cancer and melanoma, patients with increased levels of MMP-1 expression are correlated with a worse outcome regarding tumor invasion and metastasis [38]. Additionally, the MMP-1.1 2G allele may be implicated in the differentiation of gastric cancer, lymph node metastasis of breast cancer, in decreasing the age of onset in male smokers of lung cancer and tumors with infiltrative growth, and in lymph node metastases in colorectal cancer [21], [30], [42], [43]. However, stratification analyses with respect to gastric cancer progression in our study shows, those MMP-1 promoter polymorphisms are not significantly associated with invasion, lymph node metastasis, distant metastasis and thus TNM classification of gastric cancer, with some exceptional contradictions. MMP-1.3 polymorphism which shows a positive significance (*p* = 0.021) for regional lymph node metastasis having a combination of TA and TT genotype, suggests that the T allele has a detrimental effect on gastric cancer progression and early metastasis. MMP-1.4 polymorphism with a combination of the TC and CC genotype shows a negative correlation for regional lymph node metastasis in addition to distant metastasis, contradicting MMP-1.3 polymorphism. Possible explanations for this observation may reside in the degree of tumor invasion, further, metastasis in gastric carcinoma might also determined by the response to growth factors and cytokines besides the presence of 2G, G, T, C, C alleles (respectively for MMP-1.1, -1.2, -1.3, -1.4 and -1.5) in the MMP-1 promoter. Cytokines, such as interleukin-1 (IL-1), influence the expression levels of MMP-1 and act as growth stimulators correlating with liver metastasis of gastric carcinoma [44]–[46]. The MMP-1.1 polymorphism may increase the MMP-1 expression in response to growth factors and cytokines [47]. Transcriptional regulation is a complex process that is often influenced by tissue-specific factors. Although various human tumor tissues have demonstrated co-expression of Ets factor and MMP-1, there was no correlation observed between Ets expression and metastasis in pancreatic and thyroid carcinoma [48], [49]. Therefore, it is possible that MMP-1 transcriptional regulation is not directly increased by the presence of this Ets-binding site [38]. This suggests that gene expression secondary to MMP-1 SNPs is tissue specific and varies functionally between different disease processes [38]. So, the presence of polymorphic alleles of the MMP-1 promoter may not necessarily contribute to the degree of tumor invasion and in addition to progression of gastric carcinoma. However, we found a significant association between the MMP-1.2 polymorphism and the histological classification. Patients carrying at least one G allele have showed significantly greater risk for poorly differentiated (PD) carcinomas (*p* = 0.001). This finding is not observed for other SNPs in the promoter region. The histological determination of tumor grade and the subsequent clinical course of gastric cancer is subjective in nature, and more objective methods have been unsuccessfully sought to assess prognosis [38].

An increasing number of studies have shown that a disease phenotype can be associated with a linked loci or haplotype made up of polymorphisms that are not individually associated with the phenotype [50]. Functional studies showed that the MMP-1 promoter polymorphisms exert haplotype effects on MMP-1 promoter activity in cancer cells [50]. In our population, combinations of paired loci of adjacent polymorphisms do not exert any significant increased or decreased risk for gastric cancer and a gene-dose effect study confirmed the finding. Our study showed that the degree of linkage disequilibrium between the polymorphisms in the MMP-1 gene promoter is substantially low. The low degree of linkage disequilibrium between the MMP-1 gene polymorphisms likely reflects the presence of recombination hotspots at this genomic locus. Three of the five polymorphisms, i.e., MMP-1.2 (rs1144393), MMP-1.3 (rs475007) and MMP-1.4 (rs514921) have been studied in the international HapMap project, and have been shown to be located near a recombination hotspot and thus accounted for low LD [24]. We found nearly 16 haplotypes for studied MMP-1 polymorphisms, none of which contribute a major part in the population. In the MMP-1 gene promoter, in which linkage disequilibrium between polymorphisms is substantially weaker, to partition the different haplotypes would require the genotyping of four of the five polymorphisms (MMP-1.1 and MMP-1.5 are in complete linkage). Also the distribution of haplotypes of all five SNPs between patients and controls was not found to be differentially distributed, and this may be due to the possibility that individual SNPs do not exert risk for the disease. However, we have found a functional correlation of MMP-1 promoter polymorphic haplotypes with MMP-1 expression in a locoregional manner of gastric cancer occurrence. Thus it can be hypothesized that, MMP-1 promoter haplotypes may have functional importance in regulation of MMP-1 gene transcription, resulted in altered expression. This suggests MMP-1 functional polymorphic haplotypes may be a prognostic factor for locoregional gastric cancer progression.

To date, most genetic epidemiology studies of MMP-1 gene variation in relation to cancers have focused on the −1607 1G/2G polymorphism [15]–[17], [30], [31], [40], [41]. The results of our study indicate a need for genotyping additional polymorphisms in the MMP-1 gene promoter and undertaking haplotype analysis, as typing the −1607 1G/2G polymorphism alone cannot fully segregate the various MMP-1 haplotypes that may differ in promoter activity. Further studies will be required to determine the MMP-1 haplotypes and their frequencies in other populations.

In conclusion, our study suggests that MMP-1.3, MMP-1.4 and MMP-1.5 polymorphisms in the MMP-1 promoter enhances the risk of lower stomach tumor formation in an eastern Indian population. In addition, our results suggest these three adjacent polymorphisms in the MMP-1 gene promoter to be functionally important in affecting MMP-1 gene transcription, subsequently reflected in serum MMP-1 levels. Furthermore, we found that MMP-1.3 polymorphism contributed to the susceptibility of lymph node metastasis. However the current study reveals that all these three SNPs are not independent prognostic parameters to predict the outcome of patients with gastric cancer. Due to the small sample size we are unable to find any association of these polymorphisms with gastric cancer risk. Detailed epidemiological analyses of these SNPs, demographic trends and risk factors in other diseases in similar populations in addition to larger sample sizes are welcome in order to strengthen our findings. Further research may be helpful to determine whether our observations are tumor specific or applicable to other adenocarcinomas such as breast, prostate or lung cancer. Our data might serve a useful predictive parameter for early identification of individuals at-risk of lower stomach cancer and could aid strategies to optimize treatment for a disease such as gastric cancer.

## Supporting Information

File S1 Contains the following files: **Table S1:** Overall Haplotype distribution of MMP1 SNPs in lower stomach and upper stomach gastric cancer patients and association with gastric cancer risk. **Figure S1:** Genotyping of the MMP1 polymorphisms. **Figure S2:** Haplotype effect on serum MMP-1 concentration.
(DOC)
