# Supplementary material for: Matrix Metalloproteinase-1 (MMP-1) Promoter Polymorphisms are Well Linked with Lower Stomach Tumor Formation in Eastern Indian Population
Source: PLoS One. 2014 Feb 5;9(2):e88040. doi: 10.1371/journal.pone.0088040 (PMC3914871; doi:10.1371/journal.pone.0088040)
Supplement: File S1 — Contains the following files: Table S1: Overall Haplotype distribution of MMP1 SNPs in lower stomach and upper stomach gastric cancer patients and association with gastric cancer risk. Figure S1: Genotyping of the MMP1 polymorphisms. Figure S2: Haplotype effect on serum MMP-1 concentration. (DOC) [file pone.0088040.s001.doc]

**SUPPLEMENTARY TABLE:**

**Table S1: Overall Haplotype distribution of MMP1 SNPs in lower stomach and upper stomach gastric cancer patients and association with gastric cancer risk**

| **Haplotype** | **Upper Stomach** | **Lower Stomach** | **OR** | **95% CI** | **P value (a)** |
| --- | --- | --- | --- | --- | --- |
| 1G-A-A-T-T | 0.138 | 0.091 | Ref |  |  |
| 2G-A-A-T-T | 0.182 | 0.119 | 0.9915 | 0.6977-1.409 | 1 |
| 2G-A-A-T-C | 0.161 | 0.084 | 0.7912 | 0.5445-1.150 | 0.2531 |
| 2G-A-A-C-T | 0.111 | 0.113 | **1.544** | **1.064-2.241** | **0.0237** |
| 2G-A-T-T-C | 0.073 | 0.069 | 1.433 | 0.9395-2.187 | 0.1061 |
| 1G-G-A-T-T | 0.055 | 0.088 | **2.426** | **1.581-3.724** | **<0.0001** |
| 2G-A-T-C-T | 0.032 | 0.056 | **2.654** | **1.596-4.414** | **0.0002** |
| 1G-A-A-T-C | 0.065 | 0.04 | 0.9332 | 0.5806-1.500 | 0.81 |
| 1G-A-T-T-T | 0.017 | 0.068 | **6.066** | **3.350-10.985** | **<0.0001** |
| 1G-A-A-C-T | 0.017 | 0.064 | **5.709** | **3.153-10.370** | **<0.0001** |
| 2G-A-T-T-T | 0.03 | 0.039 | **1.971** | **1.143-3.399** | **0.0182** |
| 2G-G-T-C-T | 0.038 | 0.054 | **2.155** | **1.317-3.526** | **0.0028** |
| 1G-G-A-C-T | 0.02 | 0.036 | **2.73** | **1.487-5.011** | **0.0015** |
| 2G-G-A-T-C | 0.039 | 0.028 | 1.089 | 0.6262-1.893 | 0.7784 |
| 1G-G-T-C-T | 0.015 | 0.024 | **2.426** | **1.208-4.874** | **0.014** |
| 1G-A-T-T-C | 0.006 | 0.026 | **6.571** | **2.602-16.598** | **< 0.0001** |

(a) Two sided χ^2^ association P value, OR= Odds ratio, CI= Confidence Interval, Ref= Reference haplotype to calculate OR. Other possible combinations of linked loci are omitted because of null frequency in the population. Haplotype frequency and haplotype association test performed using Haploview4.2 software.

**SUPPLEMENTARY FIGURES:**

**
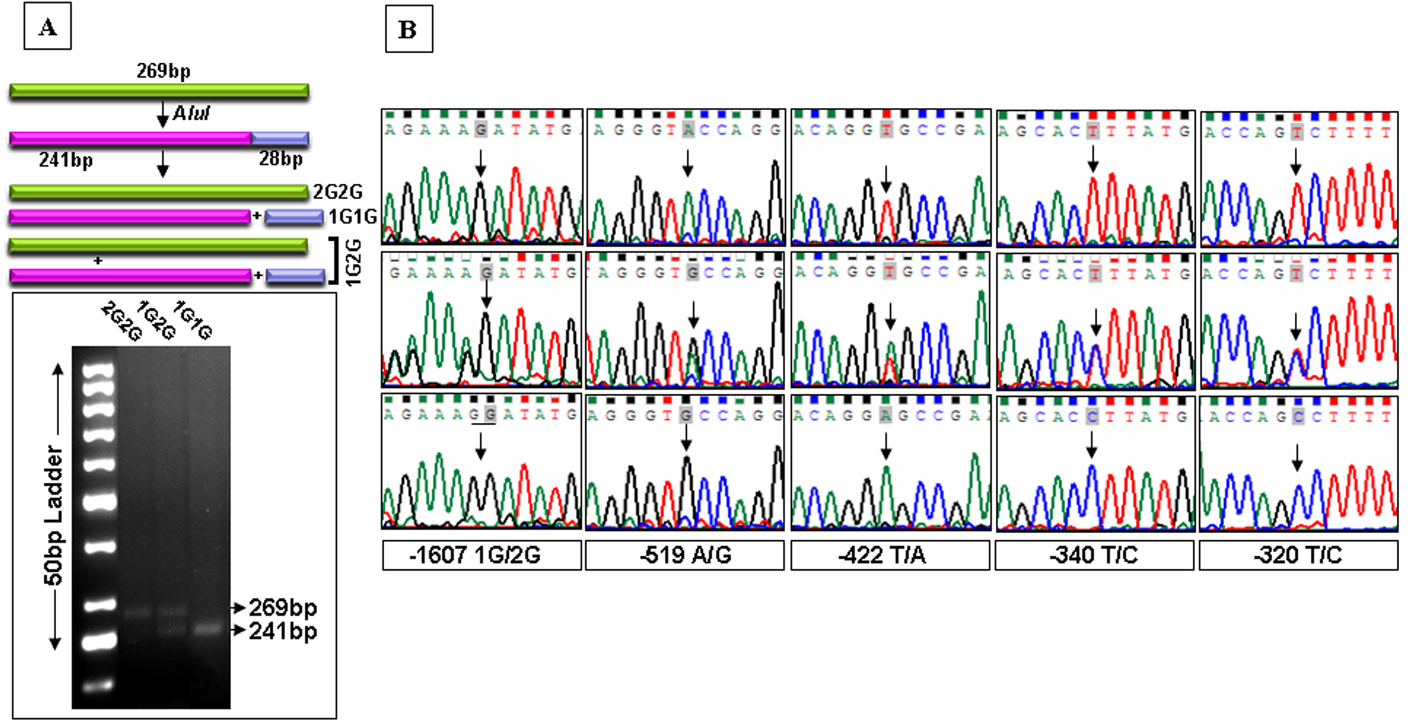
**

**Figure S1 - Genotyping of the MMP1 polymorphisms.** (A) Schematic representation of MMP1.1 RFLP analysis and representative PCR-RFLP analysis of MMP1.1 polymorphism. All possible combinations of DNA fragments resulting from PCR-RFLP are shown. 269bp target region of MMP1 gene promoter is PCR-amplified and digested with *AluI*, which cleaved the 1G allele to generate two fragments of 241bp and 28bp. 2G allele are not digested with *AluI*. The heterozygous allele shows three bands of 269bp, 241bp and 28bp; (B) Representative chromatogram of DNA sequences for each genotype for the MMP1 polymorphisms with their flanking regions. Arrows indicate the position of polymorphisms in the chromatograms.


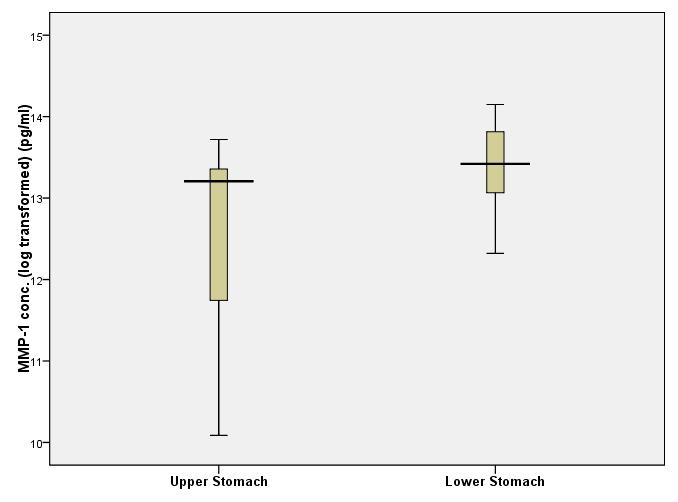


**Figure S2 – Haplotype effect on serum MMP-1 concentration**. Serum MMP-1 level in patients with lower stomach cancer with risk haplotypes (n=54), and upper stomach cancer with non risk haplotypes (n=15) were compared by ELISA. The serum MMP-1 levels were log-transformed and plotted. Results showed patients with lower stomach cancer exhibit a 1.43 fold higher MMP-1 level than in upper stomach cancer patients (p<0.05 by t-test) (box whisker diagram).
